# Management cost of respiratory syncytial virus-associated hospitalisation episodes in children: a retrospective analysis of a regionally representative medical database in Eastern China

**DOI:** 10.7189/jogh.16.04071

**Published:** 2026-03-13

**Authors:** Yumeng Miao, Xiaoyu Xu, ZhuXin Mao, Xin Wang, You Li

**Affiliations:** 1 Department of Epidemiology, National Vaccine Innovation Platform, School of Public Health, Nanjing Medical University, Nanjing, China; 2 Department of Epidemiology, School of Public Health, Key Laboratory of Public Health Safety and Emergency Prevention and Control Technology of Higher Education Institutions in Jiangsu Province, Nanjing Medical University, Nanjing, China; 3 Centre for Health Economics Research and Modelling Infectious Diseases, University of Antwerp, Antwerp, Belgium; 4 Department of Biostatistics, National Vaccine Innovation Platform, School of Public Health, Nanjing Medical University, Nanjing, China; 5 Changzhou Third People's Hospital, Changzhou Medical Centre, Nanjing Medical University, Changzhou, China; 6 Centre for Global Health, Usher Institute, University of Edinburgh, Edinburgh, UK

## Abstract

**Background:**

Respiratory syncytial virus (RSV) poses a substantial economic burden globally in young children. However, data on the economic burden of RSV are limited in low- and middle-income countries, including China. We aim to estimate the management cost of RSV-associated hospitalisations among children in China.

**Methods:**

We conducted a retrospective analysis of a regional medical database covering over 200 hospitals in Jiangsu Province, China, from 1 January 2019 to 31 May 2024. We identified RSV-associated episodes using International Classification of Diseases 10th revision codes and laboratory testing results. We reported the median (MD) and interquartile range (IQR) of direct and out-of-pocket costs per episode and analysed cost-influencing factors using multivariate linear regression.

**Results:**

The sample comprised 14 558 RSV-associated hospitalisation episodes, with the average length of stay being six days (IQR = 5–7). The average direct medical cost was USD 645 (IQR = 504–864), of which 57% (IQR = 32–76) were out-of-pocket; the highest costs were observed in infants aged <1 year (MD = USD 695; IQR = 519–933). Costs varied significantly by hospital type, with Grade III speciality hospitals having higher average costs (MD = USD 876; IQR = 705–1037). Diagnosis cost accounted for the largest proportion of the total cost (MD = 41%; IQR = 32–50), followed by medicine cost (MD = 31%; IQR = 22–41). The cost for patients with comorbidities (MD = USD 826) was 28.5% higher than that for those without comorbidities (MD = USD 643). Multivariate regression analysis revealed that younger age, comorbidities, longer length of stay, higher gross domestic product per capita in the municipality, and Grade III speciality hospitals were associated with higher direct medical costs.

**Conclusions:**

We found that RSV-associated hospitalisation costs in children are substantial in China, particularly among younger children and those with comorbidities. These findings provide critical evidence to inform RSV immunisation strategies and health-economic evaluations.

Respiratory syncytial virus (RSV) is a leading cause of severe pneumonia among young children [1]. In 2019, there were 33.0 million RSV-associated acute lower respiratory infection episodes, 3.6 million hospitalisations, and 101 400 deaths among children under five years [2]. Given the substantial disease burden of RSV, the World Health Organization Strategic Advisory Group of Experts on Immunisation recommended that all countries introduce RSV passive immunisation for infants, either through maternal vaccine or long-acting monoclonal antibodies [3].

For individual countries, the economic burden of RSV is instrumental in the decision to recommend an RSV immunisation strategy and plays an important role in the health-economic evaluation of different immunisation options in the context of vaccine-preventable diseases. Although an earlier systematic review and meta-analysis provided a global estimate of RSV-associated healthcare management costs in children under five years (approximately EUR 4.82 billion in total in 2017), the estimate was largely attributable to high-income countries [4]. By comparison, data on RSV-associated management costs were still limited in low- and middle-income countries; existing estimates were mostly based on single hospitals and thus lacked representativeness.

To address data gaps, particularly the lack of region-wide, representative cost estimates beyond single-hospital settings, we leveraged a comprehensive, regionally representative medical database from Jiangsu Province, China. This database integrated detailed management cost data with clinical and laboratory information from more than 200 hospitals across the province, providing a unique, province-wide health-system perspective. We aimed to estimate the management costs of RSV-associated hospitalisations among children under 18 years and explore the key determinants of these costs.

## METHODS

### Data source

We extracted individual patient medical records from a regional medical database that encompasses more than 200 hospitals, covering more than 95% of Grade III hospitals across all 13 municipalities in Jiangsu Province. Located in the eastern coastal region of China, Jiangsu Province is one of the most economically developed provinces, with a gross domestic product (GDP) per capita of USD 21 356 [5]; nonetheless, substantial economic disparities exist among its 13 municipalities, with southern municipalities such as Suzhou and Wuxi exhibiting higher GDP per capita compared to northern municipalities such as Suqian and Lianyungang [6]. In 2023, Jiangsu Province had a population of 85.26 million, ranking fourth among all Chinese provinces [5]. Its healthcare system is relatively developed and extensive, with approximately 563 000 hospital beds and 875 000 healthcare professionals, both core resource indicators ranking fifth nationally [7]. Detailed information on the database, as well as its data governance, storage, and processing, had been reported previously [8,9]. We conducted and reported this study in accordance with the STROBE guidelines (Table S5 in the **Online Supplementary Document**) [10].

### Study population

We included children under 18 years of age hospitalised for RSV-associated episodes between 1 January 2019 and 31 May 2024, based on their electronic hospitalisation records. For identifying RSV-associated hospitalisations, we first searched the primary diagnosis at discharge for the following International Classification of Diseases 10th revision (ICD-10) codes: J12.1 (pneumonia due to RSV), J20.5 (acute bronchitis due to RSV), J21.0 (acute bronchiolitis due to RSV), and B97.4 (RSV as the cause of diseases classified to other chapters). Since clinicians tended to use non-pathogen-specific diagnoses as a supplement to ICD-coded RSV episodes, we identified all hospitalisation records with the primary diagnosis of acute respiratory infection (ARI) at discharge (Table S1 in the **Online Supplementary Document**) and linked them to their laboratory test records using the unique inpatient identifier. We included those who tested positive for RSV, except those with a single IgG-positive serology result. We excluded a single positive IgG serology test result alone from the case definition due to its low specificity for acute infection (as IgG antibodies may persist from past exposure and do not reliably indicate the contributing agent of the current acute illness). By comparison, we included antigen and nucleic acid tests used in clinical practice because of their high specificity. Considering that children from outside the province may also seek medical treatment in Jiangsu, we strictly limited the study population to those residing in the province as identified by their admission records.

### Data collection

We collected data on basic characteristics, inpatient records, and direct medical costs. Basic characteristics included age, sex, date of birth, home address, and hospital type. There were three types of hospitals in this study: Grade III general hospitals, Grade III speciality hospitals, and Grade II hospitals; higher grades indicated higher quality of care. Inpatient records included dates of admission and discharge, primary and secondary diagnoses coded by ICD-10 (only the first two secondary diagnoses), treatment outcomes, and presence of comorbidities, which we identified based on secondary diagnoses in the discharge records; particularly, we considered the following common high-risk conditions for severe RSV diseases: congenital heart disease, bronchopulmonary dysplasia, Down syndrome, and cystic fibrosis (Table S2 in the **Online Supplementary Document**) [11]. Direct medical costs included detailed information on their components (*e.g.* hospital bed costs, examination costs, and medication costs) and on payment routes, including out-of-pocket (OOP) costs and health insurance types. In China, the two most common health insurance types were Urban Employee Basic Medical Insurance, which covered employed urban residents and their children, and Urban and Rural Residents’ Basic Medical Insurance, which covered unemployed or self-employed urban residents, rural residents, and students/children.

### Statistical analysis

We calculated median (MD) and interquartile range (IQR), as well as mean (x̄) and 95% confidence interval (CI), for the direct medical costs stratified by age group, sex, and calendar year. Length of stay, OOP cost, and its proportion in total cost were estimated and reported as MD and IQR. The direct medical costs were also aggregated by municipality, hospital type, birth month, source of admission, clinical outcome, discharge pathway, category of ARI according to the ICD-10 codes (*i.e.* acute upper respiratory infection, influenza and pneumonia, other acute lower respiratory infection, and others), comorbidities, insurance type, and RSV confirmation (*i.e.* by ICD-10 or by laboratory confirmation).

For understanding the cost-influencing factors, we plotted the relationship between length of stay and direct medical costs; as an exploratory analysis, we assessed the correlation between direct medical costs and GDP per capita at the municipality level; we also estimated the proportion of OOP costs in monthly per-capita disposable income at the municipality level. We conducted a multivariate linear regression analysis of direct medical costs with the following independent variables: age group, sex, comorbidities, length of stay, GDP per capita, and hospital type. To assess the model's robustness, we calculated variance inflation factors to detect multicollinearity.

In addition to total direct medical costs, we reported costs by category, including nursing, diagnostic (including laboratory, imaging, and clinical diagnosis), treatment, medication, and consumable costs.

All costs were adjusted for inflation to 2023 CNY using the Consumer Price Index for healthcare from the National Bureau of Statistics of China [12], and were then converted to USD using the 2023 average exchange rate (*i.e.* USD 1 = CNY 7.05). We did not apply cost discounting, as long-term economic effectiveness was not involved.

We used *R*, version 4.3.1 (R Core Team, Vienna, Austria) for all statistical analyses.

## RESULTS

### Basic characteristics

During the study period, we analysed 1 303 056 hospitalisation episodes in children under 18 years of age. Of these, 974 RSV-associated episodes were identified based on ICD-10 codes; in addition, a total of 301 383 episodes testing for RSV were identified, of which 13 584 episodes were positive for RSV, with a total of 14 558 RSV-associated hospitalisations (**Table 1**).

**Table 1:** Basic characteristics of RSV-associated hospitalisation episodes*

	Total	By ICD-10	By laboratory confirmation
**Total number**	14 558	974	13 584
**Age in years, x̄ (SD)**	2.66 (0.04)	1.59 (0.11)	2.73 (0.04)
**Age group**			
0–6 months	3080 (21.2)	351 (36.0)	2729 (20.1)
6–12 months	1866 (12.8)	178 (18.3)	1688 (12.4)
1–3 years	4295 (29.5)	289 (29.7)	4006 (29.5)
3–6 years	3883 (26.7)	128 (13.1)	3755 (27.6)
6–18 years	1434 (9.9)	28 (2.9)	1406 (10.4)
**Sex**			
Male	8163 (56.1)	559 (57.4)	7604 (56.0)
Female	6395 (43.9)	415 (42.6)	5980 (44.0)
**Municipality**			
Suzhou	2809 (19.3)	75 (7.7)	2734 (20.1)
Wuxi	514 (3.5)	250 (25.7)	264 (1.9)
Changzhou	1492 (10.2)	21 (2.2)	1471 (10.8)
Yancheng	591 (4.1)	20 (2.1)	571 (4.2)
Nanjing	932 (6.4)	75 (7.7)	857 (6.3)
Lianyungang	669 (4.6)	41 (4.2)	628 (4.6)
Huai’an	301 (2.1)	115 (11.8)	186 (1.4)
Nantong	1651 (11.3)	64 (6.6)	1587 (11.7)
Yangzhou	510 (3.5)	119 (12.2)	391 (2.9)
Zhenjiang	2323 (16.0)	6 (0.6)	2317 (17.1)
Suqian	383 (2.6)	178 (18.3)	205 (1.5)
Xuzhou	1564 (10.7)	5 (0.5)	1559 (11.5)
Taizhou	819 (5.6)	5 (0.5)	814 (6.0)
**Hospital type**			
Grade III general hospital	9025 (62.0)	688 (70.6)	8337 (61.4)
Grade III speciality hospital	5213 (35.8)	235 (24.1)	4978 (36.6)
Grade II hospital	254 (1.7)	2 (0.2)	252 (1.9)
**ARI category**			
AURI	564 (3.9)		564 (4.2)
Influenza and pneumonia	12 225 (84.0)	770 (79.1)	11 455 (84.3)
Other ALRI	1614 (11.1)	204 (20.9)	1410 (10.4)
Others	155 (1.1)		155 (1.1)
**Comorbidities**			
Yes	280 (1.9)	46 (4.7)	234 (1.7)
No	14 278 (98.1)	928 (95.3)	13 350 (98.3)
**Insurance type**			
UEBMI	831 (5.7)	144 (14.8)	687(5.1)
URRBMI	10 003 (68.8)	614 (63.0)	9389(69.1)
Others	3724 (25.6)	216 (22.2)	3508(25.8)
**Length of stay in days, MD (IQR)**	6 (5–7)	6 (5–7)	6 (5–7)

ALRI – acute lower respiratory infection, ARI – acute respiratory infection, AURI – acute upper respiratory infection, ICD – international classification of diseases, IQR – interquartile range, MD – median, RSV – respiratory syncytial virus, SD – standard deviation, UEBMI – Urban Employee Basic Medical Insurance, URRBMI – Urban and Rural Residents' Basic Medical Insurance, x̄ – mean

*Values are presented as numbers (%) unless specified otherwise.

Of the 14 558 RSV episodes, most were males (n = 8163; 56.1%), younger than three years (n = 9241; 63.5%), from Grade III general hospitals (n = 9025; 62.0%), and covered by Urban and Rural Residents’ Basic Medical Insurance (n = 10 003; 68.8%). Most of the episodes (n = 14 278; 98.1%) had no documented comorbidities. The average length of stay was six days (IQR = 5–7).

### Total direct medical costs

Overall, the average direct medical cost per episode was USD 645 (IQR = 504–864), the average OOP direct medical cost per episode was USD 362 (IQR = 201–536), accounting for 57% (IQR = 32–76) of total direct medical cost (**Table 2**). The average direct medical cost decreased with an increase in age, highest in infants aged 0–6 months (MD = USD 729; IQR = 538–967), and lowest in children aged 6–18 years (MD = USD 604; IQR = 496–758). The cost was generally comparable between males and females. There were year-on-year variations in the direct medical costs, with 2021 having the highest average cost.

**Table 2:** The direct medical cost per RSV-associated hospitalisation episode stratified by age group, sex, and year

	RSV episodes	Cost in USD		OOP cost in USD	Proportion of OOP cost
	**n**	**MD (IQR)**	**x̄ (95% CI)**	**MD (IQR)**	**MD (IQR)**
**Overall**	14 558	645 (504–864)	693 (689–697)	362 (201–536)	57 (32–76)
**Age group**					
0–6 months	3080	729 (538–967)	754 (746–763)	424 (245–675)	60 (40–100)
6–12 months	1866	649 (495–861)	689 (678–699)	379 (211–560)	59 (37–100)
1–3 years	4295	634 (498–854)	686 (679–693)	363 (194–536)	56 (31–79)
3–6 years	3883	627 (499–825)	672 (665–680)	331 (175–484)	54 (30–68)
6–18 years	1434	604 (496–758)	645 (632–657)	335 (212–430)	58 (34–66)
**Sex**					
Male	8163	650 (505–868)	696 (691–702)	364 (204–545)	57 (33–79)
Female	6395	641 (501–857)	689 (683–695)	359 (197–524)	57 (32–74)
Year*					
2020	1735	713 (531–945)	740 (729–752)	358 (125–594)	50 (24–100)
2021	4033	750 (571–968)	772 (765–780)	469 (259–643)	57 (38–100)
2022	2010	627 (484–872)	686 (675–696)	362 (0–566)	55 (0–76)
2023	5147	621 (497–795)	659 (653–666)	342 (232–462)	58 (43–68)
2024	1583	525 (434–642)	561 (549–573)	290 (187–375)	57 (32–69)

CI – confidence interval, IQR – interquartile range, MD – median, OOP – out of pocket, RSV – respiratory syncytial virus, x̄ – mean

*Not including 2019, as no data on laboratory confirmation were available.

The total direct medical cost (MD = USD 876; IQR = 705–1037) and the proportion of OOP cost (MD = 60%; IQR = 49–100) were the highest in Grade III specialty hospitals (**Table 3**). Among different ARI categories, influenza and pneumonia cases had the highest average direct medical costs (MD = USD 673; IQR = 529–888), whereas acute upper respiratory infections had the lowest (MD = USD 445; IQR = 373–551). The average direct medical cost was significantly higher among patients with comorbidities (MD = USD 826; IQR = 600–1022) than among those without (MD = USD 643; IQR = 502–860). While those covered by Urban Employee Basic Medical Insurance tended to have higher direct medical costs, the average OOP cost was the lowest. The RSV episodes confirmed by ICD-10 codes (MD = USD 556; IQR = 451–719) had lower average total direct medical cost than those identified through laboratory-testing records (MD = USD 654; IQR = 509–875).

**Table 3:** Total direct medical cost per RSV-associated hospitalisation episode stratified by multiple factors*

	RSV episodes, n	Total cost in USD	OOP cost in USD	Proportion of OOP cost
**Type of hospital**				
Grade III general hospital	9025	566 (466–698)	292 (134–426)	54 (25–72)
Grade III speciality hospital	5213	876 (705–1037)	513 (373–754)	60 (49–100)
Grade II hospital	254	482 (404–563)	258 (215–317)	51 (41–70)
**Birth month**				
January	1232	636 (501–851)	352 (174–520)	57 (30–73)
February	1090	629 (500–837)	353 (214–508)	57 (33–72)
March	1167	636 (500–832)	351 (186–510)	56 (30–74)
April	1046	641 (510–842)	365 (213–542)	58 (37–75)
May	1029	637 (497–863)	361 (196–520)	56 (31–75)
June	1104	639 (502–861)	363 (202–527)	56 (33–76)
July	1205	651 (505–857)	359 (172–536)	55 (30–75)
August	1346	655 (503–876)	375 (207–545)	57 (36–77)
September	1279	661 (511–899)	358 (192–565)	56 (30–80)
October	1382	652 (512–889)	371 (223–547)	58 (37–79)
November	1418	653 (503–876)	363 (204–561)	59 (34–100)
December	1260	654 (506–870)	369 (225–556)	58 (39–81)
**Source of admission**				
Emergency	2072	641 (511–805)	322 (170–478)	53 (29–72)
Outpatient	12 347	648 (503–877)	367 (210–543)	58 (36–77)
Transfer	105	560 (488–691)	416 (180–569)	71 (30–100)
Others	34	610 (544–668)	302 (112–360)	53 (13–65)
**Clinical outcome**				
Recovered	2475	599 (497–736)	291 (12–424)	52 (2–69)
Improved	12 029	662 (507–891)	378 (227–561)	58 (40–80)
Not improved	44	536 (420–735)	303 (13–470)	60 (2–75)
Others	10	782 (538–944)	285 (153–480)	46 (24–62)
**Discharge pathway**				
Discharge on medical advice	14 323	646 (505–866)	362 (202–536)	57 (33–76)
Transfer on medical advice	14	590 (447–652)	220 (166–365)	47 (28–60)
Transfer to a community hospital on medical advice	55	646 (507–775)	136 (0–267)	21 (0–42)
Discharge against medical advice	157	573 (437–795)	377 (241–582)	68 (46–100)
Others	9	683 (389–882)	311 (242–333)	36 (32–75)
**ARI category**				
AURI	564	445 (373–551)	258 (0–380)	60 (0–74)
Influenza and pneumonia	12 225	673 (529–888)	376 (217–557)	57 (36–76)
Other ALRI	1614	530 (430–704)	298 (103–440)	57 (22–87)
Others	155	591 (439–818)	322 (172–473)	60 (37–75)
**Comorbidity**				
CHD	266	820 (599–1022)	472 (330–838)	67 (48–100)
Other comorbidities	14	938 (733–1005)	426 (245–838)	55 (33–94)
No	14 278	643 (502–860)	360 (199–532)	57 (32–76)
**Insurance type**				
UEBMI	831	649 (530–782)	179 (0–326)	30 (0–49)
URRBMI	10 003	623 (495–826)	331 (192–457)	53 (30–66)
Others	3724	732 (538–960)	611 (363–900)	100 (58–100)
**RSV confirmation**				
By ICD-10	974	556 (451–719)	281 (152–409)	54 (27–68)
By laboratory confirmation	13 584	654 (509–875)	368 (205–543)	57 (33–77)

ALRI – acute lower respiratory infection, ARI – acute respiratory infection, AURI – acute upper respiratory infection, CHD – congenital heart disease, ICD – international classification of diseases, OOP – out of pocket, RSV – respiratory syncytial virus, UEBMI – Urban Employee Basic Medical Insurance, URRBMI – Urban and Rural Residents' Basic Medical Insurance

*Values are presented as median (interquartile range).

### Influencing factors of direct medical costs

**Figure 1:**
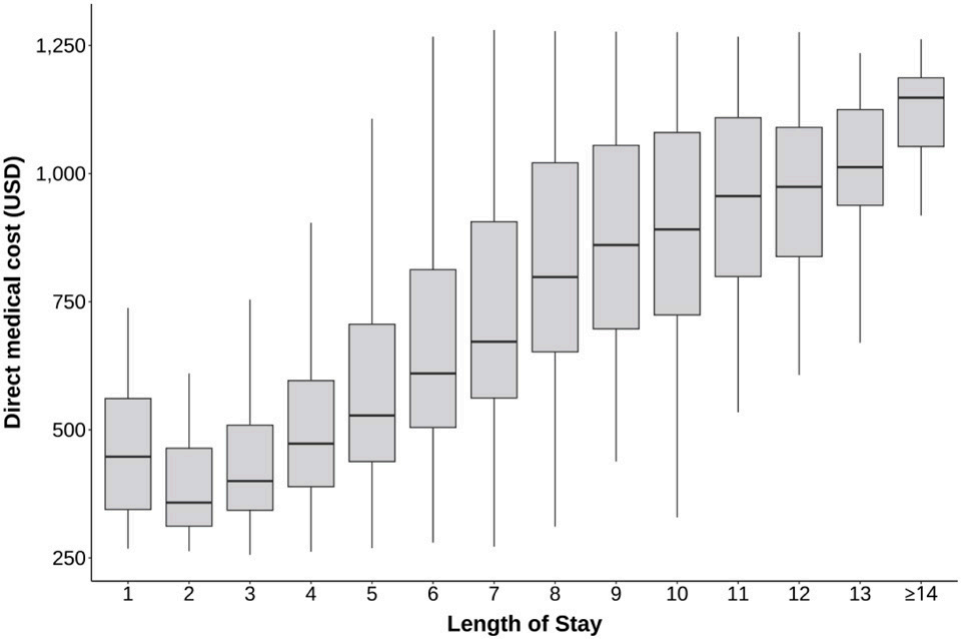
Direct medical costs of RSV-associated hospitalisation by length of stay. The box represents the interquartile range (IQR) with the median line inside the box. The whiskers (lines extending from the box) represent the range of the data within 1.5 times the IQR from the quartiles. RSV – respiratory syncytial virus.

**Figure 2:**
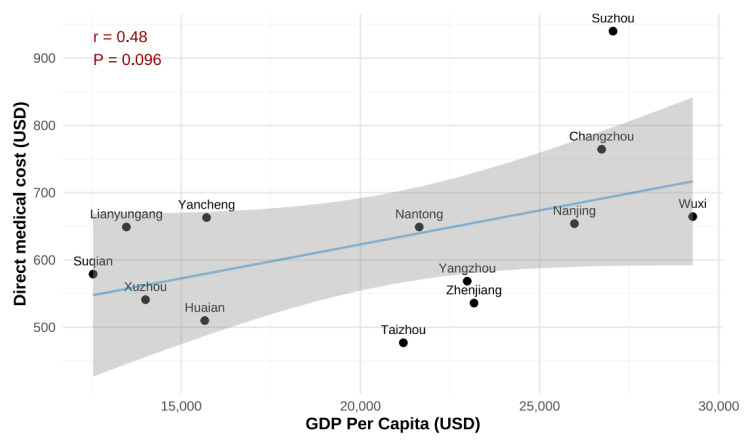
Correlation between GDP per capita and median direct medical cost of RSV-associated hospitalisation at municipality level. GDP – gross domestic product, RSV – respiratory syncytial virus.

Length of stay was positively correlated with the average total direct medical cost (*r* = 0.51) (**Figure 1**). There were substantial variations in the direct medical costs among the 13 municipalities of the province, with higher costs observed in municipalities with higher GDP per capita, although the correlation was not statistically significant (**Figure 2**). The average proportion of OOP cost in per capita monthly disposable income ranged from 39% (IQR = 26–53) in Nanjing to 86% (IQR = 17–117) in Yancheng (Table S3 in the **Online Supplementary Document**).

**Figure 3:**
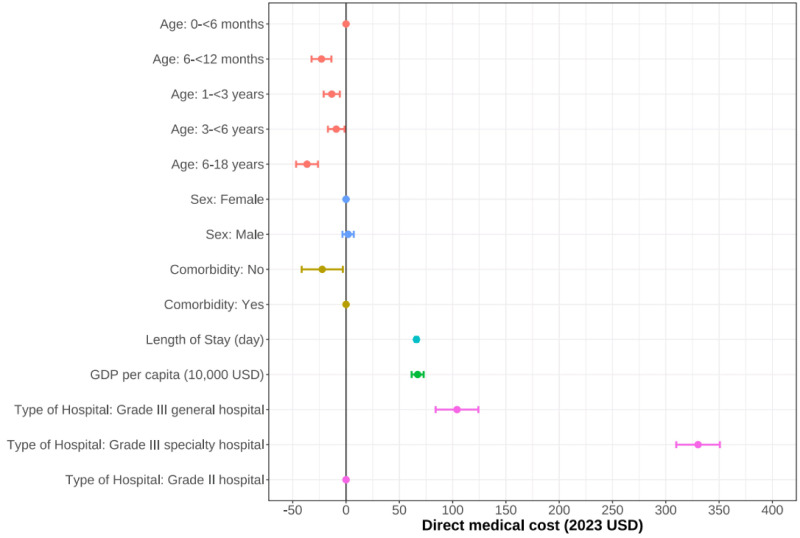
Results of multivariate linear regression analysis of total direct medical costs. GDP – gross domestic product.

Results from the multivariate regression analysis showed consistent findings with the descriptive analysis above. Younger age, comorbidities, longer length of stays, higher GDP per capita, and Grade III speciality hospitals were associated with higher direct medical costs (**Figure 3**). Model diagnostics confirmed the appropriateness of the linear regression specification, with no evidence of severe multicollinearity (all variance inflation factors <2).

### Direct medical costs by category

Diagnosis costs alone accounted for more than 40% of total direct medical costs (MD = USD 248; IQR = 175–390), primarily laboratory testing (MD = USD 219; IQR = 156–328). Medicine costs were the second-highest, accounting for 31% (IQR = 22–41) of the total direct medical costs (Table S4 in the **Online Supplementary Document**)

## DISCUSSION

By leveraging a large, regionally representative medical database, we analysed the management costs of more than 14 000 RSV-associated hospitalisations across four RSV seasons. The findings highlighted the substantial economic cost of RSV-associated hospitalisations in children in China, particularly among younger children and children with comorbidities. China has licensed nirsevimab for use in infants, and other prophylactic products such as clesrovimab were licensed in the USA [13]. Our estimates address an important gap in the economic burden of paediatric RSV disease, thereby supporting cost-effectiveness evaluations of future RSV immunisation strategies.

We found that the average direct medical cost per RSV-associated hospitalisation episode was USD 645, consistent with previous reports from a smaller sample in China [14–16]. For instance, a study conducted between 2005 and 2009 in Suzhou, China, reported an average cost of USD 572 per RSV-associated hospitalisation among 2721 RSV-positive hospitalised children, with an average length of stay of eight days [14]. However, a single-hospital-based study from Henan Province, China, reported a much higher cost despite a comparable length of stay; Ren *et al.* [17] estimated that among 261 RSV-positive inpatient children under five years, the average direct medical cost during hospitalisation was USD 1065, suggesting that there could be large variations across hospitals and regions. We found that even within the same province, substantial variation remained across the 13 municipalities, with a weak positive correlation between GDP per capita and total direct medical costs, suggesting that regions with higher levels of economic development tend to incur higher costs associated with RSV hospitalisations. In addition to geographical variations, we found that costs varied by hospital type, with average costs reaching USD 876 in Grade III speciality hospitals, the same hospital type as in Ren’s study [17]. However, this disparity should be interpreted with caution, as it likely reflects a combination of factors beyond hospital type alone. Higher costs in speciality hospitals may be driven by higher case severity (*e.g.* receiving referrals of complex patients), increased utilisation of advanced diagnostic and therapeutic technologies, and distinct institutional pricing structures.

Although our estimated direct medical cost was substantially lower than that reported in other high-income Asian countries, such as Japan (approximately USD 3300 with a comparable length of stay) [18], these estimates should be interpreted in the context of the local disposable income and OOP costs. At the municipality level, we found that OOP costs accounted for more than 80% of the monthly disposable income in three municipalities, suggesting that RSV-related medical costs impose a substantial economic burden on households. This was also the case in many other low- or middle-income countries; in Bangladesh, the average OOP cost for RSV-related hospitalisation represented 24% of the monthly household income, and over 50% families borrowed money to meet the medical cost [19]. The variation in OOP burden by health insurance type was expected, given the differing reimbursement rates. For example, the Urban Employee Basic Medical Insurance has the highest reimbursement rate (*e.g.* approximately 90% for employees hospitalised in Grade III hospitals in Nanjing) and thus has the lowest OOP burden.

Notably, a peak in average direct medical costs was observed in 2021 (USD 750), possibly due to a slightly longer average length of stay (*i.e.* seven days) in that year compared with other years (*i.e.* six days).

We found that the average direct medical cost in children with documented comorbidities was over 25% higher than that of those without comorbidities, consistent with previous studies [4,20–23], highlighting the importance of targeted interventions for high-risk groups. However, it is worth noting that over 95% of the RSV-associated hospitalisation episodes in this study occurred in children without documented comorbidities, indicating that RSV infection affected not only children with underlying conditions but also primarily otherwise healthy children [18].

There were some limitations in our study that should be acknowledged. First, as a retrospective study, we relied on existing medical records from the database, which were not originally collected for research purposes. In particular, the included RSV cases were those diagnosed by clinicians or confirmed by a clinician-ordered RSV test; however, RSV tests used in clinical settings were not 100% sensitive. Therefore, our study was subject to selection bias if the cost of management differed between RSV-infected children with recorded RSV infections and those without. Second, our identification of comorbidities was based on the first two secondary diagnoses, which may not capture all underlying conditions, particularly in children with multiple comorbidities. However, the overall prevalence of documented comorbidities in our study (1.9%) is consistent with previous reports from similar settings in China (*e.g.* approximately 2% prevalence of congenital heart disease in Suzhou) [14], suggesting that the impact of this limitation on our overall cost estimates is likely modest. Third, although our study is by far one of the largest studies reporting RSV management cost in children and covers a wide range of hospitals, the study region (Jiangsu Province, China) might not fully represent the entire country of China since the access, affordability, and quality of healthcare could vary substantially across regions. Nonetheless, the municipality-level analysis in this study provided initial evidence of regional variation in RSV management costs and their determinants, such as GDP per capita. Additionally, our cost assessment was limited to direct medical costs during hospitalisation. We did not capture direct non-medical costs (*e.g.* transportation) or indirect costs (*e.g.* loss of income due to caregivers' absence from work). Moreover, we did not include the costs of outpatient or self-medication prior to admission. Consequently, our estimates should be interpreted as a conservative lower bound of the true socioeconomic burden of paediatric RSV hospitalisation. The already high OOP expenditures we observed, accounting for an average of 57% of direct medical costs, highlight a significant financial risk to families. If other RSV-associated costs are considered, such as direct medical costs (*e.g.* outpatient care), direct non-medical costs (*e.g.* transportation), and indirect costs (*e.g.* loss of productivity), the financial burden on families would be even more pronounced. Finally, our analysis was restricted to children whose primary diagnosis was ARI to ensure that the medical cost could be primarily attributable to ARI rather than other coexisting conditions. Nonetheless, we could not rule out the presence of co-infections (*e.g.* bacteria) and their contribution to the cost. Therefore, the true RSV-attributable cost could be lower than what we estimated.

## CONCLUSIONS

Leveraging a large, regionally representative medical database, we quantified the direct medical economic burden of RSV-associated hospitalisations among children in China. The findings show that RSV infection imposes a substantial economic pressure on the healthcare system, with a high OOP proportion representing a substantive burden on household disposable income. These detailed, real-world cost estimates provide crucial baseline parameters for future health-economic evaluations of RSV immunisation strategies (*e.g.* using nirsevimab or other prophylactic products) and are instrumental for informing cost-effective public health intervention policies.

## Additional material


Online Supplementary Document
