# Supplementary material for: Management cost of respiratory syncytial virus-associated hospitalisation episodes in children: a retrospective analysis of a regionally representative medical database in Eastern China
Source: J Glob Health. 2026 Mar 13;16:04071. doi: 10.7189/jogh.16.04071 (PMC12981738; doi:10.7189/jogh.16.04071)
Supplement: Online Supplementary Document [file jogh-16-04071-s001.pdf]

Supplement to: Miao Y, Xu X, Mao Z, Wang X, Li Y. Management cost of respiratory syncytial virus-associated hospitalisation episodes in children: a retrospective analysis of a regionally representative medical database in Eastern China. J Glob Health. 2026;16:04071.

Table of Contents

Table S1. ICD-10 codes for identifying acute respiratory infection 2
Table S2. ICD-10 codes for identifying selected comorbidities 2
Table S3. Median OOP cost, GDP per capita and disposable income per capita at the municipality level 3
Table S4. Category-specific cost of RSV-associated hospitalisation 4

Table S1. ICD-10 codes for identifying acute respiratory infection.

**Sub-category**	**ICD-10 codes**
AURI	J00-J06
Influenza and pneumonia	J09-J18
Other ALRI	J20-J22
Others	
Bacterial, viral and other infectious agents	B95-B97
Other bacterial diseases	A40-A41,A49

AURI – acute upper respiratory infection; ALRI = acute lower respiratory infection.

Table S2. ICD-10 codes for identifying selected comorbidities.

**Comorbidity**	**ICD-10 codes**
Congenital heart disease	Q20-Q28
Bronchopulmonary dysplasia	P27.1
Down syndrome	Q90.9
Cystic fibrosis	E84 (E84.0, E84.9)

Table S3. Median OOP cost, GDP per capita and disposable income per capita at the municipality level.

**Municipality**	**Median OOP cost in USD (IQR)**	**GDP per capita (USD)**	**Disposable income per capita/year (USD)**	**Median proportion (%) of OOP cost in monthly disposable income (IQR)**
Nanjing	329 (219–448)	25,972	10,233	39 (26–53)
Wuxi	417 (288–715)	29,273	9,794	51 (35–88)
Xuzhou	276 (156–505)	14,004	5,405	61 (35–112)
Changzhou	360 (195–649)	26,731	8,882	49 (26–88)
Suzhou	558 (436–821)	27,049	10,512	64 (50–94)
Nantong	316 (202–438)	21,642	7,358	52 (33–71)
Lianyungang	341 (271–401)	13,470	5,106	80 (64–94)
Huai’an	296 (225–440)	15,657	5,543	64 (49–95)
Yancheng	417 (82–573)	15,707	5,854	86 (17–117)
Yangzhou	277 (163–355)	22,981	6,772	49 (29–63)
Zhenjiang	313 (0–403)	23,170	7,885	48 (0–61)
Taizhou	329 (225–489)	21,199	6,990	57 (39–84)
Suqian	329 (0–405)	12,542	4,639	85 (0–105)

GDP – gross domestic product, IQR – interquartile range, OOP – out of pocket, USD – United States dollar

Table S4. Category-specific cost of RSV-associated hospitalisation.

Cost Category	Median (IQR) (USD)	Percentage of the total cost (%)
Nursing	45 (36–56)	7 (5–9)
Diagnosis	248 (175–390)	41 (32–50)
Laboratory diagnosis	219 (156–328)	37 (28–46)
Other diagnosis	14 (2–41)	2 (0–6)
Medicine	208 (125–309)	31 (22–41)
Consumable	22 (11–36)	3 (2–6)

IQR – interquartile range, RSV – respiratory syncytial virus, USD – United States dollar

STROBE Statement—Checklist of items that should be included in reports of ***cross-sectional studies***

	Item No	Recommendation	Page No
**Title and abstract**	1	(*a*) Indicate the study's design with a commonly used term in the title or the abstract	P1
		(*b*) Provide in the abstract an informative and balanced summary of what was done and what was found	P1-2
**Introduction**			
Background/rationale	2	Explain the scientific background and rationale for the investigation being reported	P2-3
Objectives	3	State specific objectives, including any prespecified hypotheses	P3
**Methods**			
Study design	4	Present key elements of study design early in the paper	P3-4
Setting	5	Describe the setting, locations, and relevant dates, including periods of recruitment, exposure, follow-up, and data collection	P3-4
Participants	6	(*a*) Give the eligibility criteria, and the sources and methods of selection of participants	P4
Variables	7	Clearly define all outcomes, exposures, predictors, potential confounders, and effect modifiers. Give diagnostic criteria, if applicable	P4-5
Data sources/ measurement	8*	For each variable of interest, give sources of data and details of methods of assessment (measurement). Describe comparability of assessment methods if there is more than one group	P4-6
Bias	9	Describe any efforts to address potential sources of bias	P4
Study size	10	Explain how the study size was arrived at	P4
Quantitative variables	11	Explain how quantitative variables were handled in the analyses. If applicable, describe which groupings were chosen and why	P5-6
Statistical methods	12	(*a*) Describe all statistical methods, including those used to control for confounding	P5-6
		(*b*) Describe any methods used to examine subgroups and interactions	P5
		(*c*) Explain how missing data were addressed	NA
		(*d*) If applicable, describe analytical methods taking account of sampling strategy	NA
		(*e*) Describe any sensitivity analyses	NA
**Results**			
Participants	13*	(a) Report numbers of individuals at each stage of study—eg numbers potentially eligible, examined for eligibility, confirmed eligible, included in the study, completing follow-up, and analysed	P6
		(b) Give reasons for non-participation at each stage	NA
		(c) Consider use of a flow diagram	NA
Descriptive data	14*	(a) Give characteristics of study participants (eg demographic, clinical, social) and information on exposures and potential confounders	P6
		(b) Indicate number of participants with missing data for each variable of interest	NA
Outcome data	15*	Report numbers of outcome events or summary measures	P7
Main results	16	(*a*) Give unadjusted estimates and, if applicable, confounder-adjusted estimates and their precision (eg, 95% confidence interval). Make clear which confounders were adjusted for and why they were included	P8

		(*b*) Report category boundaries when continuous variables were categorized	P7
		(*c*) If relevant, consider translating estimates of relative risk into absolute risk for a meaningful time period	NA
Other analyses	17	Report other analyses done—eg analyses of subgroups and interactions, and sensitivity analyses	P7
**Discussion**			
Key results	18	Summarise key results with reference to study objectives	P8
Limitations	19	Discuss limitations of the study, taking into account sources of potential bias or imprecision. Discuss both direction and magnitude of any potential bias	P10-11
Interpretation	20	Give a cautious overall interpretation of results considering objectives, limitations, multiplicity of analyses, results from similar studies, and other relevant evidence	P8-11
Generalisability	21	Discuss the generalisability (external validity) of the study results	P10-11
**Other information**			
Funding	22	Give the source of funding and the role of the funders for the present study and, if applicable, for the original study on which the present article is based	P12

*Give information separately for exposed and unexposed groups.

Note: An Explanation and Elaboration article discusses each checklist item and gives methodological background and published examples of transparent reporting. The STROBE checklist is best used in conjunction with this article (freely available on the Web sites of PLoS Medicine at http://www.plosmedicine.org/, Annals of Internal Medicine at http://www.annals.org/, and Epidemiology at http://www.epidem.com/). Information on the STROBE Initiative is available at www.strobe-statement.org.
